# Comprehensive Machine Learning-Based Model for Predicting Compressive Strength of Ready-Mix Concrete

**DOI:** 10.3390/ma14051068

**Published:** 2021-02-25

**Authors:** Jiajia Xu, Li Zhou, Ge He, Xu Ji, Yiyang Dai, Yagu Dang

**Affiliations:** Department of Chemical Engineering, Sichuan University, Chengdu 610065, China; xujiajia@stu.scu.edu.cn (J.X.); chezli@scu.edu.cn (L.Z.); hegescu@gmail.com (G.H.); daiyy@scu.edu.cn (Y.D.); derkdang@scu.edu.cn (Y.D.)

**Keywords:** ready-mix concrete, compressive strength, random forest, feature selection, genetic algorithm

## Abstract

Considering that compressive strength (CS) is an important mechanical property parameter in many design codes, in order to ensure structural safety, concrete CS needs to be tested before application. However, conducting CS tests with multiple influencing variables is costly and time-consuming. To address this issue, a machine learning-based modeling framework is put forward in this work to evaluate the concrete CS under complex conditions. The influential factors of this process are systematically categorized into five aspects: man, machine, material, method and environment (4M1E). A genetic algorithm (GA) was applied to identify the most important influential factors for CS modeling, after which, random forest (RF) was adopted as the modeling algorithm to predict the CS from the selected influential factors. The effectiveness of the proposed model was tested on a case study, and the high Pearson correlation coefficient (0.9821) and the low mean absolute percentage error and delta (0.0394 and 0.395, respectively) indicate that the proposed model can deliver accurate and reliable results.

## 1. Introduction

Concrete has long been the most widely used building material all over the world due to its multifold merits in integrity, durability, modularity and economy. According to the development report of China’s ready-mix concrete industry in 2020, the production in the first three quarters reached 1.94 billion cubic meters. Among the various performance indices of concrete, compressive strength (CS) is the most important, as it directly affects the building’s structural safety.

Traditionally, the CS of concrete is obtained by testing specifically prepared and cured cubic or cylindrical specimens using a compression test instrument, which is cumbersome, time-consuming and costly in the entire experimental process. To improve this situation, empirical regression methods [1,2] and numerical simulation [3,4] have been developed to predict concrete CS based on the design recipe. The concrete production process is affected by several factors, which have strong nonlinear relationships with the product CS and are strongly interrelated. With the rapid development of machine learning, there is a trend to employ data-driven techniques for concrete CS prediction. Compared with the conventional regression methods, machine learning-based approaches adopt suitable algorithms to automatically “learn” from the process data, “distinguish” important influential factors from the interfering factors, approximate the intricate process mechanism with deterministic mathematical forms, and perform prediction with high accuracy over a specified confidence interval. To date, various machine learning algorithms have been applied to studying the correlations between the concrete recipe and the product CS. Sobhani et al. [5] constructed both traditional regression models and machine learning models to predict the 28-day CS of no-slump concrete, based on the concrete ingredients (including the amount of cement, silica fume, water, coarse aggregates, fine aggregates, and fillers). They found that the machine learning models were more feasible than the traditional models. Chou et al. [6], Al et al. [7], and Cheng et al. [8,9] exploited several machine learning techniques to study the high nonlinear relationships between the ingredients and high-performance concrete CS. Besides the basic ingredients for conventional concrete, the authors also considered supplementary cementitious materials, including fly ash, blast-furnace slag and chemical admixtures. Aiyer et al. [10] examined the capability of least-squares support vector machine (SVM) and relevance vector machine for the determination of self-compacting concrete CS, and concluded that the latter can deliver robust prediction results. Behnood et al. [11] applied the M5P model tree for the CS prediction of normal concrete and high-performance concrete based on the amount of concrete constituent. Recently, Yu et al. [12] developed an optimized self-learning method for the CS prediction of high-performance concrete. Feng et al. [13] employed the adaptive boosting algorithm to construct a strong learner by integrating several weak learners, to enhance the predictive accuracy. Their model also considered the influence of the curing time in addition to the concrete mixture components.

The aforementioned methods are based on the assumption that the quality of raw materials is stable, which in most cases is not true, especially when construction and demolition wastes (i.e., recycled aggregate concrete) and manufactured sand concrete are used. Considering this, several machine learning algorithms have been applied to predict the CS of concrete built from various types and sources of aggregates as raw materials, including artificial neural networks (ANNs) [14,15,16,17,18] and enhanced support vector regression (SVR) [19]. Dantas et al. [14] developed an ANN to predict the CS of concrete containing construction and demolition waste, in which aggregate quality was added as input. Multiple studies have confirmed the impact of aggregate quality on concrete CS [16,17,19,20,21]. In addition to aggregates, the properties of cementitious materials, such as CS and tensile strength, have also been proposed as variables to predict the concrete CS [22].

Researchers have also proved that besides the type and basic properties of raw materials, environmental factors, such as temperature [23,24], and relative humidity [25], also significantly determine the concrete CS. To exclude the influence from these environmental factors, Atici [26] outlined the need of fixing the ambient temperature and relative humidity when comparing the prediction performances of different methods. Additionally, the recipe and environmental variables may not be sufficient in covering all the possible factors influencing concrete CS. It has been proved in many areas that to comprehensively evaluate the quality of an engineering product, the influences from man, material, machine, method and environment (shortened to 4M1E), should be considered [27,28,29,30,31]. Each of these factors further represents the aggregation of various detailed influential factors. For example, the “man” aspect includes the comprehensive capacity of project participants, covering the leadership ability of managers, the technical ability of direct operators, and the understanding of quality supervisors.

The combined effect of the above factors (4MIE) poses new challenges for the accurate prediction of concrete CS. Since the influence from the “material” aspect is already inherently complicated, when the joint influences from the other four aspects are included, the problem dimension becomes high, and it will be more difficult to manually identify the most relevant factors from the interfering ones. Computer-aided feature selection techniques provide an alternative solution to this situation. Common feature selection algorithms can be categorized into two classes based on the search strategies—the filter methods and the wrapper methods. Dantas et al. [14], and Ly et al. [22] applied principal component analysis (PCA) to reduce the noise in the input space and consequently improve the model predictive performance. Principal component analysis is a typical filter method that performs feature selection based on the statistical performance of the original dataset and is independent of the subsequent learning algorithm. Different from the filter methods, the wrapper methods tightly couple the subsequent prediction algorithm with the feature selection process [32]. In other words, the feature selection process is optimized based on the feedback from the subsequent algorithm performance. Therefore, wrapper methods usually have a better learning effect than filter methods [33,34]. Commonly used wrapper methods include genetic algorithms (GAs) [35] and particle swarm optimization (PSO) [36].

This paper proposes a machine learning-based predictive model that integrates a genetic algorithm (GA) and random forest (RF) to comprehensively evaluate the various influencing factors from different aspects, aiming to accurately predict concrete CS. First, influential factors from five perspectives (i.e., man, machine, material, method, and environment) are collected to support a comprehensive evaluation of the concrete production process. Second, GA is applied to perform feature selection automatically based on the predictive performance of the subsequent modeling algorithm. In this way, disturbance variables can be adaptively eliminated, and the best model prediction accuracy can be achieved. Third, RF is used to correlate the selected process features to the concrete CS. Given the characteristics of the concrete production process, RF is considered a very suitable modeling algorithm, due to its versatile merits, such as its good tolerance for outliers and noises, its ability to avoid overfitting, and its ability to deal with multicollinearity [37,38].

## 2. Research Significance

The main contributions of this research can be divided into two points. First, to the best of our knowledge, so far there is no work that focuses on the prediction of concrete CS considering the comprehensive process features (4M1E). The introduction of the 4M1E quality management concept takes into account every influencing factor as much as possible, so that the proposed model fills the lack of reliable prediction models of early concrete CS in actual production. Second, GA is applied to automatically select suitable features for concrete production modeling. The combination of multi-dimensional influencing factors (4M1E) increases the complexity of manual identification of the most relevant variables from interference variables. For this problem, adaptive feature selection performed by GA can effectively eliminate redundant variables and improve model prediction accuracy.

## 3. Data Collection and Pre-Processing

To support comprehensive evaluation, production process factors influencing concrete CS are systematically collected from five perspectives—man, machine, material, method and environment (Table 1). First, the “man” factors reveal the comprehensive capacity of the concrete production participants, who can indirectly affect the concrete quality by impacting the material, the machine, the method used, and the environment. In this work, three indicators are used to study the influence of the personnel aspect: the work shifts ($a_{1}$), age ($a_{2}$), and seniority ($a_{3}$). Second, as the indispensable tool for production, the machine directly affects the concrete property. In this study, the considered impact from the machine aspect mainly covers the reliability of the material weighing scales and the stable current value of the mixing unit. The former directly affects the real constituent of the final concrete product and is quantitatively characterized by the measurement deviation, while the latter reflects the resistance fluctuation of the material mixing process and is represented by the value of stable current. Equation (1) calculates the measurement deviation for the material weighing scales, wherein positive values indicate an overdose of the corresponding raw material, and negative values denote an underdose.

(1) $$deviation=\frac{x_{actual}-x_{plan}}{x_{plan}}\times 100\%$$

where $x_{actual}$ and $x_{plan}$ are the actual and planned weighted values of raw materials, respectively.

Third, the “material” factors mainly refer to the quality and the content of materials used. The material constituents included in “material” part are—ordinary Portland cement, slag powder, fly ash, fine stone, gravel, fine sand, coarse sand, tap water, recycled water, superplasticizer and expansive agent. The considered material quality includes the CS of cement ($c_{2}$), the fineness of fly ash ($c_{8}$), the aggregate water-content ($c_{15}$), and the fineness modulus of coarse sand ($c_{20}$). To eliminate the influence of different production batches, the raw material consumption amount is converted into percentage based on the overall raw material consumption amount. Fourth, the “method” factors in this study consider the engineering design for the concrete production, including the water-to-cement ratio (W/C), sand ratio (SR), and design strength. The following equations give the calculation formulas for W/C and SR:

(2) $$W/C=\frac{c_{22}+c_{23}}{c_{1}}$$

(3) $$SR=\frac{c_{16}+c_{19}}{c_{11}+c_{13}}$$

where $c_{1}$, $c_{22}$ and $c_{23}$ denote the contents of cement, tap water, and recycled water, respectively; $c_{11}$ and $c_{13}$ represent the contents of fine stone and gravel, respectively; $c_{16}$ and $c_{19}$ represent the contents of fine sand and coarse sand, respectively.

Finally, the “environment” factors considered are temperature (including the minimum temperature, maximum temperature and average temperature) and relative humidity.

To sum up, 45 process features reflecting impacts from the five engineering production aspects (4M1E) were collected to comprehensively evaluate and accurately predict the corresponding concrete CS. The actual concrete CS is measured by the compressive testing of a cube sample with a height of 150 mm. For method validation, 321 datasets were collected from the production process of ready-mix concrete of an enterprise in Southeast China. The data sample was small. Table 2 presents the profile of the collected data. Detailed information is provided in the Supplementary Materials.

For convenience, the collected process features from the five aspects were combined into one vector by following a certain order, and the *j*th individual process feature recorded for concrete production batch *i* is denoted as $x_{i,j}$. Thus, the process features collected for concrete CS modeling can be expressed as a matrix *X*, which is of dimension d×m.

(4) $$\begin{array}{c} X=\left[\begin{array}{ccccc} x_{1,1} & \cdots & x_{1,j} & \cdots & x_{1,m}\\ \vdots & \ddots & \vdots & \ddots & \vdots \\ x_{i,1} & \cdots & x_{i,j} & \cdots & x_{i,m}\\ \vdots & \ddots & \vdots & \ddots & \vdots \\ x_{d,1} & \cdots & x_{d,j} & \cdots & x_{d,m} \end{array}\right] \end{array}$$

where *m* denotes the number of all the collected process features, and *d* represents the number of data entries collected from different concrete production batches. Here, *m* equals 45. Each row of the matrix, denoted as $X_{i}$, represents the process features collected for concrete production batch *i*.

(5) $$\begin{array}{c} X_{i}=\left[x_{i,1},\ldots ,x_{i,j},\ldots ,x_{i,m}\right] \end{array}$$

and,

(6) $$\begin{array}{c} X={\left[X_{1},\ldots ,X_{i},\ldots ,X_{d}\right]}^{T} \end{array}$$

Meanwhile, the actual concrete CS for production batch *i* is denoted as $y_{i}$, and the collection of all the production batches is denoted as *Y*. Then, the predicted variable can be expressed as a matrix of dimension 1×d.

(7) $$\begin{array}{c} Y={\left[y_{1},\ldots ,y_{i},\ldots ,y_{d}\right]}^{T} \end{array}$$

Dataset noise, incomplete data entry, and outliers were removed to ensure model accuracy. Furthermore, the processed data were mapped to the scale of [0,1] based on the following equation to eliminate the effect of dimensional difference:

(8) $$x_{j}^{\prime }=\frac{x_{j}-{\left(x_{j}\right)}_{\min}}{{\left(x_{j}\right)}_{\max}-{\left(x_{j}\right)}_{\min}}$$

The collected datasets [X, Y] were then proportionally divided into a training set (matrix size: 257×45) and a test set (matrix size: 64×45) at 4:1 for the later stage of model training and model validation, respectively.

## 4. The Proposed GA-RF Methodology for Concrete CS Prediction

**Figure 1:**
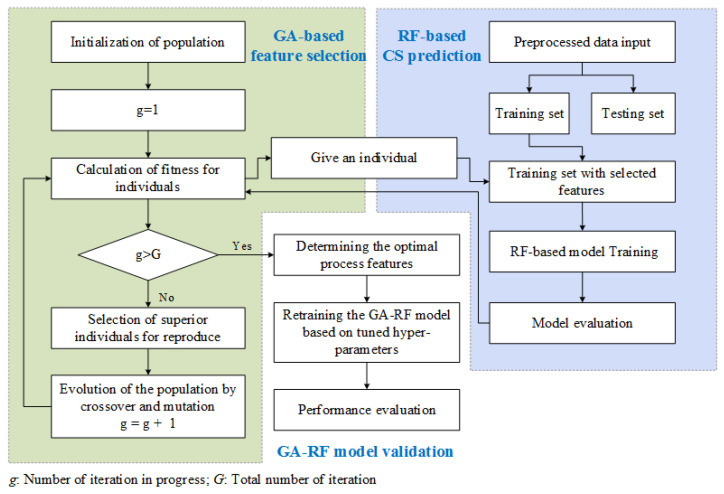
Flowchart of genetic algorithm-random forest (GA-RF) for the compressive strength (CS) prediction.

In this section, a hybrid machine-learning-based method is introduced for the prediction of concrete CS. The flowchart of the proposed method is illustrated in Figure 1. As is shown, the method is composed of two collaborative modules—a GA-based feature selection and an RF for the concrete CS modeling and prediction. The modeling process is described in detail in the following subsections.

### 4.1. RF for Concrete CS Prediction

Random forest is an ensemble learning algorithm that has been extensively applied in complex engineering problems due to its capability in dealing with outliers, noises, and multicollinearity and avoiding overfitting. In this study, RF was adopted to solve the regression problem of concrete CS prediction. This section briefly introduces the formulations for the model development of concrete CS prediction. More details on RF can be found in Appendix A. All the selected process features were used as inputs for concrete CS modeling by RF. For regression problems, the final prediction result was performed by averaging the outputs of all trees. This process is represented as follows:

(9) $$\tilde{Y}=\frac{1}{n}\sum_{k=1}^{n}{\tilde{Y}}_{k}=\frac{1}{n}\sum_{k=1}^{n}f_{k}\left(X\right)$$

(10) $$\begin{array}{c} \tilde{Y}={\left[{\tilde{y}}_{1},\ldots ,{\tilde{y}}_{i},\ldots ,{\tilde{y}}_{d}\right]}^{T} \end{array}$$

where ${\tilde{Y}}_{k}$ represents the output of the *k*th decision tree model $f_{k}\left(X\right)$, *n* denotes the number of decision trees; and ${\tilde{y}}_{i}$ denotes the predictive CS of production batch *i*.

### 4.2. GA for Feature Selection

After the inclusion of the various influential factors from the five different aspects (4M1E), the problem of concrete CS prediction becomes much more complex than the original one, since some of those factors may or may not play important roles in determining the CS of the corresponding product concrete. The existence of redundant and irrelevant process features not only increases the computational complexity, but also decreases the accuracy of the predictive model. Thus, important process features must be identified and trivial ones must be excluded. In this section, a GA-based feature selection procedure is introduced for the selection of critical features of concrete production process. The GA is suitable for optimization problems with both continuous and discrete functions and can efficiently search huge and complex optimization spaces. Regarding the GA application in feature selection, Table 3 presents an analogy between biological terms and feature selection.

#### 4.2.1. Initialization of Population

The GA is a population-based optimization algorithm. In this work, each individual in the population corresponds to a subset of the collected process features. For example, the *p*th individual is denoted as follows:

(11) $$B_{p}=\left[b_{j}\right]\;j=\left\{1,2,\ldots ,m\right\}$$

subject to

(12) $$b_{j}=\left\{\begin{array}{cc} 0 & \mathrm{exclusion}\;\mathrm{of}\;\mathrm{the}\;\mathrm{corresponding}\;\mathrm{process}\;\mathrm{feature},\\ 1 & \mathrm{inclusion}\;\mathrm{of}\;\mathrm{the}\;\mathrm{corresponding}\;\mathrm{process}\;\mathrm{feature}. \end{array}\right.$$

**Figure 2:**
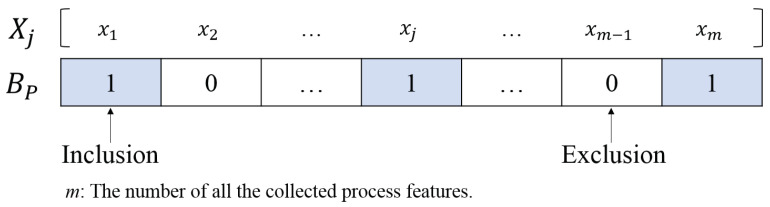
Representation of an individual as a binary string.

Figure 2 illustrates the relationship between a process feature collection $X_{j}$ and an individual $B_{p}$. Population initialization was performed by randomly generating a number of individuals.

#### 4.2.2. Calculation of Fitness for Individuals

After the population initialization, the corresponding variable sets were used as inputs for RF modeling. The input for RF modeling was determined by the following equation, where $S_{p}$ represents the selected process features for the *p*th individual:

(13) $$S_{p}=B_{p}\times X$$

Subsequently, the selected process features determined by each population individual served as input variables for concrete CS model training by the RF.

After the model training process, each of the obtained RF were evaluated. In this paper, $R^{2}$ was selected as the fitness function for each population individual to assess the predictive accuracy of the obtained RF model. The larger the fitness, the higher the model accuracy. The fitness is calculated by the following formula:

(14) $$\begin{array}{c} R^{2}=1-\frac{\sum {\left(Y-{\tilde{Y}}_{p}\right)}^{2}}{\sum {\left(Y-\bar{Y}\right)}^{2}} \end{array}$$

where ${\tilde{Y}}_{p}$ denotes the output of the RF model corresponding to the *p*-th individual and $\bar{Y}$ denotes the average of *Y*.

#### 4.2.3. Termination Condition Evaluation

The termination of the feature selection process was determined by generation information of individuals. If the number of evolution generations *G* is met, the fifth step follows; otherwise, the process goes to the next step.

#### 4.2.4. Evolution of the Population

After the fitness assessment for the individuals in a generation, if the termination condition was yet to be met, individuals of the current generation then underwent a series of evolution processes, including the selection of superior individuals, crossover and mutation, to produce populations of a new generation with high diversity. In this study, the roulette wheel selection method was used to select individuals with superior performance. The selection process is based on a calculated probability by the following formula:

(15) $$pl\left(B_{p}\right)=\frac{f(B_{p})}{\sum f(B_{p})}$$

where $f(B_{p})$ is the calculated value of the *p*-th individual in the population. To maintain population diversity, random sampling with replacement was adopted. The same number of individuals as the primary population were selected as parents for the next step.

The crossover and mutation operations were performed based on two predefined probabilities. In this study, the crossover probability and mutation probability were set to 0.7 and 0.2, respectively.

### 4.3. GA-RF Model Validation

After *G* generations of evolution, the best individual $B_{p}^{*}$ was obtained; that is, the CS forecast model with the optimal feature subset was determined. The GA-RF model was then retrained based on the tuned hyper-parameters by a grid-search algorithm. The testing set was then used for model validation.

To better evaluate the performance of the proposed model, four commonly used statistical parameters were used.

Pearson correlation coefficient (R) is widely used to measure the statistical relationship between two variables. The closer the value is to 1, the better the model fits. The mathematical expression of *R* is as follows:

(16) $$R=\frac{N\sum y_{i}\cdot {\tilde{y}}_{i}-\left(\sum y_{i}\right)\left(\sum {\tilde{y}}_{i}\right)}{\sqrt{N\left(\sum y_{i}^{2}\right)-{\left(\sum y_{i}\right)}^{2}}\cdot \sqrt{N\left(\sum {{\tilde{y}}_{i}}^{2}\right)-{\left(\sum {\tilde{y}}_{i}\right)}^{2}}}$$

where *N* is the number of data points.

Mean absolute error (MAE) and Root mean squared error (RMSE) are used to describe the differences between predictive values and the actual values. For a good fit, their value should be close to zero. Compared with MAE, RMSE gives outliers more weight by squaring to amplify deviation. Therefore, RMSE is more sensitive to outliers and reflects the variation of error; MAE is more robust to outliers, and better reflects the real situation of predicted value errors. MAE and RMSE are calculated by the following equation:

(17) $$MAE=\frac{1}{N}\sum \left|y_{i}-{\tilde{y}}_{i}\right|$$

(18) $$RMSE=\sqrt{\frac{1}{N}\sum {\left|y_{i}-{\tilde{y}}_{i}\right|}^{2}}$$

In order to better represent the magnitude of the prediction error change, delta is introduced to represent the difference between RMSE and MAE. The smaller the delta, the more stable the prediction result [39].

(19) Δ=RMSE−MAE

Mean absolute percentage error (MAPE) uses the percentage of error relative to the actual value to measure the accuracy. Compared with MAE and RMSE, MAPE is equivalent to normalizing the error of each point, reducing the influence of the absolute error caused by individual outliers. The smaller the MAPE value, the smaller the relative overall error. Calculation formula for MAPE is as follows:

(20) $$MAPE=\frac{1}{N}\sum \left|\frac{y_{i}-{\tilde{y}}_{i}}{y_{i}}\right|$$

## 5. Results and Discussion

**Figure 3:**
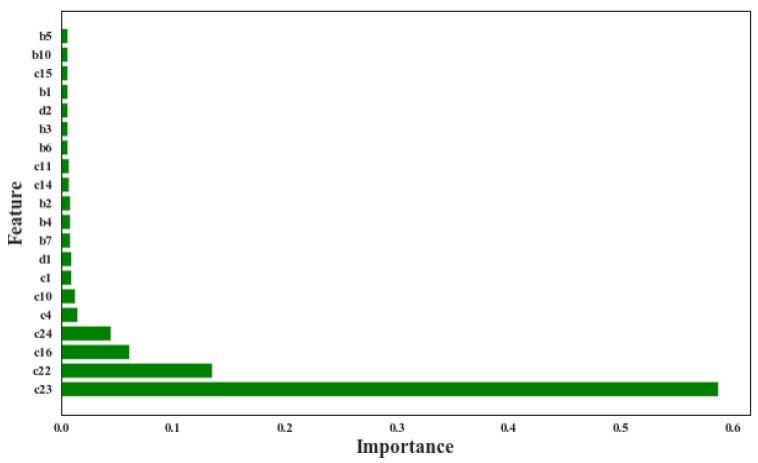
Variable importance of concrete CS measured by using RF model.

The collected process features were analyzed and the concrete CS prediction model was developed based on the analysis results. This section presents the analysis and modeling results. Random forest was used to assess the importance of each collected process feature to the concrete CS, and the evaluation result is partially given in Figure 3. The top seven most important factors all come from the “material” aspect, implying that the material ingredients are the most critical impact factors for concrete CS. Also, $c_{10}$ (the activity index of fly ash) ranks as the sixth important factor, suggesting that the quality of materials is not trivial for high-accuracy prediction models.

It is worth reminding that some specific feature rankings will vary with different enterprises. In this case, recycled water content ($c_{23}$) ranking first is in line with the actual production of the studied enterprise. In response to the call of environmental protection, the company usually adopts the method of partial or complete recovery of wastewater to achieve the goal of zero discharge of slurry water as much as possible. Recycled water can affect the mechanical properties and microstructure of concrete [40,41]. Due to different sources and random consumption of recycled water, its composition and content vary greatly. Compared with the precise control of cement content ($c_{1}$) and water-cement ratio ($d_{1}$), the large fluctuation of recycled water has the most obvious impact on the CS of concrete, followed by tap water used for supplementation. Therefore, in order to better control the concrete CS, it is recommended to use tap water alone or other water with small fluctuations.

**Figure 4:**
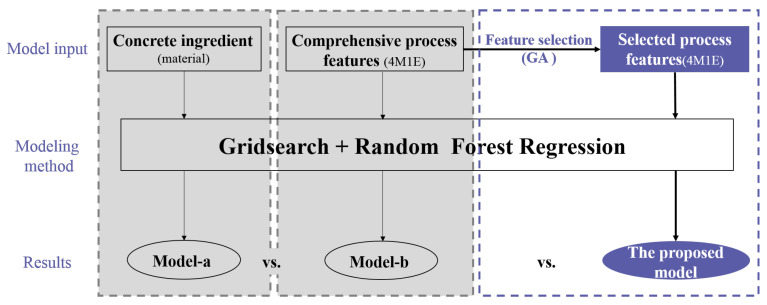
Framework of results discussion.

Although the importance of the factors from other production aspects is not so remarkable, they may be nonnegligible for the enhancement of process model accuracy. To verify this conjecture, three modeling approaches were applied to a case study—a traditional method that takes the concrete ingredients as the only input features for RF modeling (model-a); a comprehensive modeling method that considers all the collected influential factors from the five aspects (4M1E) as input features for RF modeling (model-b); and the proposed modeling methodology, which integrates RF modeling with a feature selection process (the proposed model). As shown in Figure 4, the results derived from these three models were compared and discussed in the following section.

### 5.1. Result Comparison between the Concrete Ingredient Modeling and the Comprehensive 4M1E Modeling

**Figure 5:**
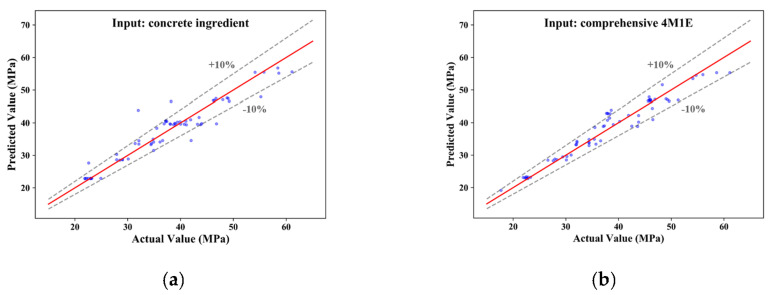
Comparison between the recorded actual concrete CS and the predicted value from: (**a**) concrete ingredient modeling, and (**b**) comprehensive 4M1E modeling.

**Figure 6:**
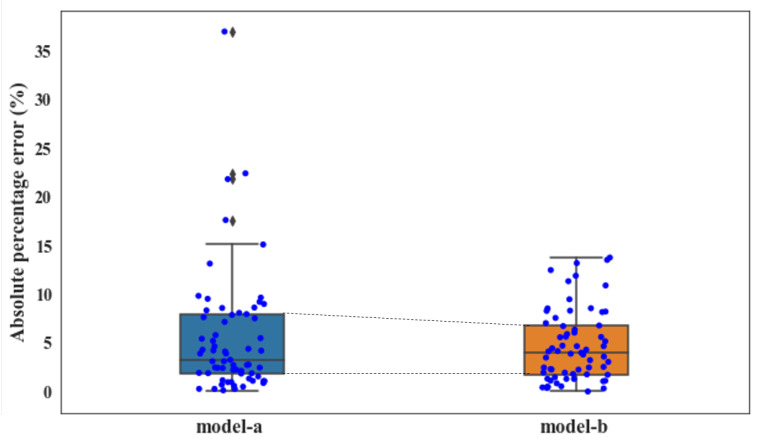
Boxplot of error on testing set obtained by model-a and model-b.

Figure 5 compares the recorded actual concrete CSs and the predicted values from model-a and model-b. For both cases, most of the predictive concrete CS values are closely distributed along the diagonal lines, implying that the RF algorithm is suitable for the concrete CS modeling and prediction. Additionally, several predictive values fell out of the ±10% error range when only concrete ingredients were considered in the modeling; this phenomenon is significantly improved in the case of model-b, where comprehensive modeling is performed based on influential factors from the 4M1E aspects. Furthermore, regarding the predictive performance of these two models, model-b presented a remarkably higher R (0.9707 vs. 0.9564), and much lower MAE (1.846 vs. 2.081), RMSE (2.428 vs. 3.079), and MAPE (0.0475 vs. 0.0551) (Table 4). The lower delta value (0.582 vs. 0.998) also suggests that the model-b performance based on a more comprehensive evaluation is more stable and reliable. This can be further confirmed by the visualization of the calculated prediction error of the two models. As illustrated by the boxplots in Figure 6, a narrower interquartile range with relatively smaller upper quartile and fewer outliers were observed from model-b; moreover, the median of the boxplot of model-a is closer to the bottom of the box, which means that the calculated errors above the median value are more dispersed.

**Figure 7:**
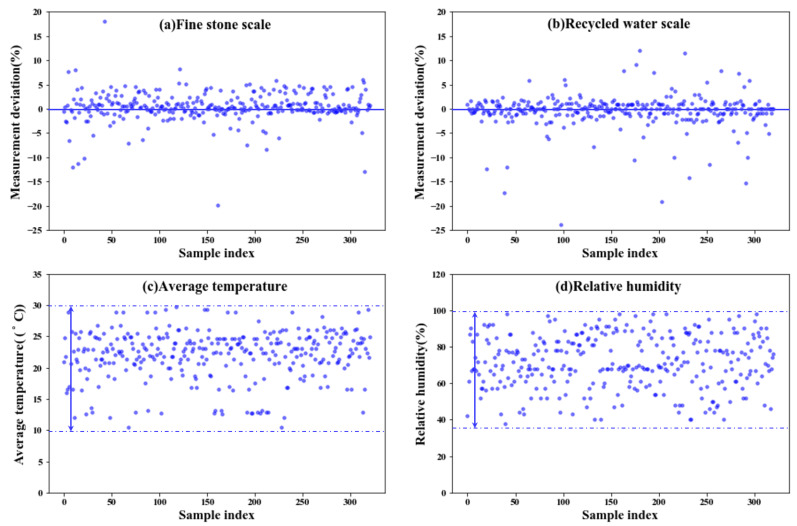
Scatter plot of some influencing factors of concrete, (**a**) fine stone scale; (**b**) recycled water scale; (**c**) average temperature; (**d**) relative humidity.

The reasons for the above results are proposed as follows—the development of model-a is based on the following assumptions: (1) concrete ingredients are the key factors affecting the CS, which is true according to the data analysis results shown in Figure 3 and (2) other possible influential factors from the production process (such as man, machine, method, and environment) remain fixed. However, in most cases, this is not true. For example, in most of the production processes, operators shift according to certain working schedules. The difference in the comprehensive capacity of direct operators can indirectly affect the concrete quality by impacting material, machine, method, and environment. Also, due to mechanical and/or aging reasons, the performances of machines are usually not stable. As shown in Figure 7, the measured weight for fine stone (Figure 7a) and recycled water (Figure 7b) and the expected targeting weight usually do not match. In some cases, the deviation even exceeds 10% and is close to 20%, which will directly affect the actual consumption of these materials. As for the environmental factors, they are usually time-dependent variables according to the local climate. Figure 7c,d show the average temperature and relative-humidity distribution of the collected datasets for the case study. The average temperature spans between 10 °C and 30 °C, and the relative humidity falls in the range of 38% to 100%.

### 5.2. Result Comparison between the Comprehensive 4M1E Modeling and 4M1E Modeling with Feature Selection

The previous section discusses the necessity of performing comprehensive modeling based on influential factors from 4M1E, to improve the model accuracy. This section addresses the need for proper feature selection for further improving the model performance.

**Figure 8:**
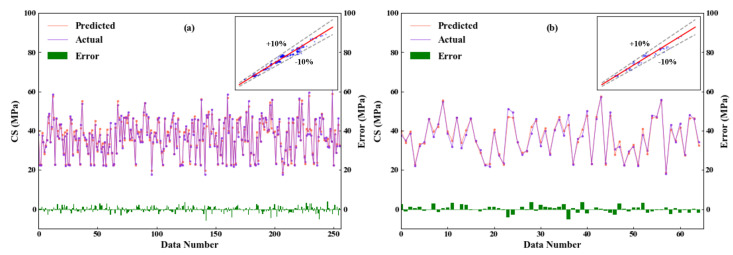
Comparison between the recorded actual concrete CS and the predicted value on the training set (**a**) and testing set (**b**) from the proposed GA-RF modeling.

**Figure 9:**
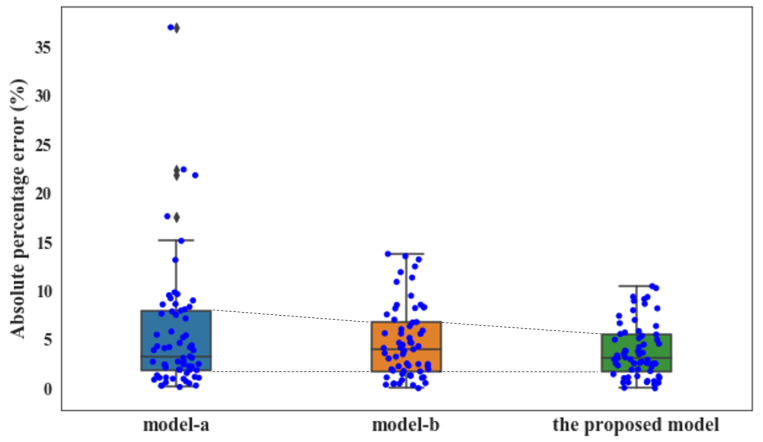
Boxplot of error on testing set obtained by different inputs.

**Figure 10:**
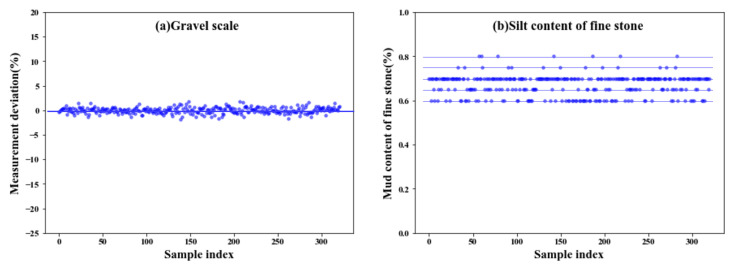
Data distribution of two of the excluded unimportant process factors: (**a**) weighting error of gravel weighting scale, and (**b**) the silt content of fine stones.

Although the inclusion of the influential factors from the other four production aspects significantly enhanced the model accuracy, it also increased the problem dimension and thus the computational burden. Additionally, interfering factors may have been introduced. To further improve the predictive accuracy, the proposed modeling approach with the GA integrated as the automatic feature selection algorithm was applied. Figure 8 compares the recorded actual concrete CS and the predicted value from the proposed GA-RF modeling. For both the training and testing sets, the predicted values are evenly distributed alongside the diagonal line and fall within the ±10% error range. The slight errors between the predicted values and actual concrete CS depicted on the horizontal axis indicate the high prediction accuracy of the proposed GA-RF model. Table 5 compares the predictive performance among model-a (modeling with the concrete ingredient), model-b (modeling with comprehensive factors from 4M1E), and the proposed model (modeling with feature selection from 4M1E factors). The proposed model presented the highest R (0.9821) and the least MAE, RMSE, and MAPE (1.429, 1.824, and 0.0394, respectively) implying that the predictive accuracy of the obtained model with feature selection was the highest. Furthermore, the stability and reliability of the obtained model were also improved, as seen from the decreased delta (0.395). To better compare the model performances, the calculated prediction errors of all three models are visualized in Figure 9 via boxplots. As is shown, after a feature selection procedure, the interquartile range was further reduced by a smaller upper quartile and almost no outliers occurred. This is because among the influential factors outside the “material” aspect, some important ones affect the concrete CS, such as the fluctuating process factors shown in Figure 7; there are also insignificant ones that do not significantly affect the concrete CS, but instead will interfere with other factors in the modeling process. A feature selection process can identify the important ones and exclude the trivial ones. For example, Figure 10 illustrates the data distribution of two of the excluded unimportant process factors, including the weighting error of the gravel weighing scale and the silt content of fine stones. As can be seen, the fluctuation magnitude of the weighting error for gravel is rather small, and the silt content of fine stone is roughly distributed on several fixed levels. This is because, in this case, the silt content of the fine stone is obtained from human estimations, rather than from experiments, implying that the quality of this data item is considerably influenced by the engineering experience of the direct operators (man). A low data accuracy will interfere with the model accuracy; therefore, it is better to exclude redundant, disturbing data from the modeling procedure.

### 5.3. Comparison with Other Methodologies

This section compares the proposed approach and several other feature selection and modeling methods to assess the competitiveness of the proposed method.

The comparison involves the following feature selection approaches—PCA and grey relational analysis (GRA) and the following process modeling methods—multiple linear regression (MLR), ANN, and SVR. The results are presented in Table 6.

As shown in the first row of the table, through the application of RF for process modeling, a relatively high predictive accuracy was reached. Compared with the other three modeling methods, ANN was not suitable for this modeling task. Moreover, the model accuracy and the model reliability of ANN combined with the GA feature selection procedure (GA-ANN) were not as satisfactory as those of RF alone. The proposed method (GA-RF), that is RF integrated with the GA feature selection procedure, reduced the process features (20 vs. 44 for RF alone) and improved the model accuracy to 0.9821. Additionally, compared with RF and the combinations of the GA and the other three modeling methods, the GA-RF presented a reduced delta value, indicating enhanced model reliability. Hence, RF is more suitable for the modeling and prediction of concrete CS. This is because the sampling approach and voting mechanism of the RF algorithm prevent the model from overfitting and can reduce noise interference. Based on these results, compared with ANN, SVR, and MLR, RF might be more suitable for predicting concrete production process data because of its better resistance to interference. Furthermore, the GA outperformed the other two feature selection algorithms by increasing the model accuracy from 0.9707 to 0.9821. Although PCA could significantly reduce the process feature (from 44 to 9), which implies reduced computational burden of the modeling process, the model accuracy was not satisfactory. For GRA, it could remove the redundant process feature and slightly enhance the model accuracy and reliability, but the effectiveness was not as encouraging as that of the GA.

## 6. Conclusions

To establish a reliable concrete CS prediction model and thus reduce the time-consuming and laborious laboratory tests, this study investigated the influencing factors of concrete CS from the perspective of quality management; moreover, a GA-RF forecasting model considering process factors from five process aspects, including man, machine, material, method and environment, as the model inputs is proposed. A GA was applied to perform feature selection adaptively based on the predictive performance of the subsequent modeling algorithm and RF was used to correlate the selected process features to the concrete CS. The proposed method was applied to the production of an actual ready-mix concrete enterprise in Southeast China, and the results proved its applicability and effectiveness. The following conclusions were derived: (1) A comprehensive process-evaluation from related five engineering aspects (man, machine, material, method, and environment), rather than considering only for factors from the “material” aspect, can improve the model accuracy; (2) an automatic process feature selection procedure can not only alleviate modelers’ burden but also effectively reduce the model complexity and improve the model accuracy; (3) compared with ANN, SVR, and MLR, RF is more competent in the modeling of concrete production process.

In conclusion, the proposed approach performs comprehensive system modeling by considering influencing factors from five engineering aspects and automatically eliminating process disturbance variables. As a methodology, it provides the possibility of individualized and refined quality management for various concrete enterprises. It can be extended to the modeling and prediction of tensile strength, slump, and other properties of concrete. Early determination of the mechanical properties of concrete is very important for concrete technology and civil engineering. On the one hand, it checks whether the concrete strength meets the requirements and gives feedback to guide the production process; on the other hand, the early determination of the concrete strength assists the engineer in the safety design analysis during the construction phase to reduce lag. However, further efforts are still required. For example, some of the process features (model inputs) will shift over time; thus, the time threshold needs to be further discussed. Also, more extensive datasets need to be collected to more comprehensively describe the production process and further improve the generalization ability of the GA-RF model.

## Figures and Tables

**Table 1:** Influential process factors for concrete production from five perspectives.

Input	Variable	Unit	Variable	Unit
Man	*a${}_{1}$*: work shifts	-	*a${}_{2}$*: age	year
	*a${}_{3}$*: seniority	month		
Machine	*b${}_{1}$*: gravel scale	%	*b${}_{2}$*: coarse sand scale	%
	*b${}_{3}$*: fine stone scale	%	*b${}_{4}$*: powder scale 1 ${}^{1}$	%
	*b${}_{5}$*: powder scale 2	%	*b${}_{6}$*: additives scale 1	%
	*b${}_{7}$*: additives scale 2	%	*b${}_{8}$*: tap water scale	%
	*b${}_{9}$*: recycled water scale	%	*b${}_{10}$*: stable current value	A
Material	*c${}_{1}$*: cement content ${}^{2}$	-	*c${}_{2}$*: compressive strength of cement	MPa
	*c${}_{3}$*: cement paste fluidity	mm	*c${}_{4}$*: slag powder content	-
	*c${}_{5}$*: fluidity ratio	-	*c${}_{6}$*: activity index of slag powder	%
	*c${}_{7}$*: fly ash content	-	*c${}_{8}$*: fineness of fly ash	%
	*c${}_{9}$*: water demand ratio of fly ash	%	*c${}_{10}$*: activity index of fly ash	%
	*c${}_{11}$*: fine stone content	-	*c${}_{12}$*: silt content of fine stone	%
	*c${}_{13}$*: gravel content	-	*c${}_{14}$*: silt content of gravel	%
	*c${}_{15}$*: aggregate water-content	%	*c${}_{16}$*: fine sand content	-
	*c${}_{17}$*: fineness modulus of fine sand	-	*c${}_{18}$*: silt content of fine sand	%
	*c${}_{19}$*: coarse sand content	-	*c${}_{20}$*: fineness modulus of coarse sand	-
	*c${}_{21}$*: silt content of coarse sand	%	*c${}_{22}$*: tap water content	-
	*c${}_{23}$*: recycled water content	-	*c${}_{24}$*: superplasticizer content	-
	*c${}_{25}$*: expansive agent content	-		
Method	*d${}_{1}$*: water-cement ratio	-	*d${}_{2}$*: sand ratio	-
	*d${}_{3}$*: design strength	MPa		
Environment	*e${}_{1}$*: Minimum temperature	${}^{\circ }C$	*e${}_{2}$*: maximum temperature	${}^{\circ }C$
	*e${}_{3}$*: average temperature	${}^{\circ }C$	*e${}_{4}$*: relative humidity	%

^1^ The measuring range of weighing scale 1 is generally larger than that of weighing scale 2. ^2^ The material content is a percentage without unit.

**Table 2:** A brief illustration of the collected input data from the five engineering aspects and the output.

	Variable	Unit	No				Range
			1	2	...	321	
Input	*a${}_{1}$*	-	0	1	...	1	0, 1
	...	...	...	...	...	...	...
	*a${}_{3}$*	month	60	14	...	24	12–60
	*b${}_{1}$*	%	−0.095	0.2083	...	0.0417	−1.80–1.84
	...	...	...	...	...	...	...
	*b${}_{10}$*	A	64.94	65.67	...	69.81	35.1–76.3
	*c${}_{1}$*	-	0.1305	0.1533	...	0.1407	0.07–0.17
	...	...	...	...	...	...	...
	*c${}_{25}$*	-	0.0153	0.016	...	0.016	0–0.016
	*d${}_{1}$*	-	0.6071	0.4396	...	0.4278	0.41–1.14
	...	...	...	...	...	...	...
	*d${}_{3}$*	MPa	50	45	...	40	15–50
	*e${}_{1}$*	${}^{\circ }C$	19.4	19.9	...	22.8	5.9–27.5
	...	...	...	...	...	...	...
	*e${}_{4}$*	%	85	78	...	68	38–98
Output	*s*	MPa	49.4	45.8	...	43.5	17.7–61.1

**Table 3:** Common terms in GA versus biology.

Terms in Biology	Genetic Algorithm (GA) in Feature Selection
Gene	Binary variables indicating the inclusion or exclusion of a process feature from the following five aspects (man, machine, material, method, environment)
Chromosome	A vector consists of the whole set of genes for the concrete production process
Crossover	Exchange of variables between two parent individuals at randomly selected sites
Mutation	Change the selection state of variables at randomly selected sites
Fitness	Performance evaluation of RF model

**Table 4:** Predictive performance comparison of model-a and model-b.

Input	Performance Measures				
	R	MAE(MPa)	RMSE(MPa)	MAPE	Δ
model-a	0.9564	2.081	3.079	0.0551	0.998
model-b	0.9707	1.846	2.428	0.0475	0.582

**Table 5:** Predictive performance comparison of the three obtained models.

Input	Performance Measures				
	R	MAE(MPa)	RMSE(MPa)	MAPE	Δ
model-a	0.9564	2.081	3.079	0.0551	0.998
model-b	0.9707	1.846	2.428	0.0475	0.582
the proposed model	0.9821	1.429	1.824	0.0394	0.395

**Table 6:** Predictive performance of different model on the testing set.

Model	Performance Measures					Feature-Selected
	R	MAE (MPa)	RMSE (MPa)	MAPE	Δ	
RF	0.9707	1.846	2.428	0.0475	0.528	44
GA-ANN	0.9619	2.155	2.686	0.0631	0.531	21
GA-SVR	0.9708	1.919	2.327	0.0541	0.408	18
GA-MLR	0.9723	1.821	2.246	0.0521	0.425	20
GA-RF	0.9821	1.429	1.824	0.0394	0.395	20
PCA-RF	0.9167	2.587	3.862	0.0797	1.275	9
GRA-RF	0.9724	1.835	2.343	0.0468	0.508	17

**Figure A1:**
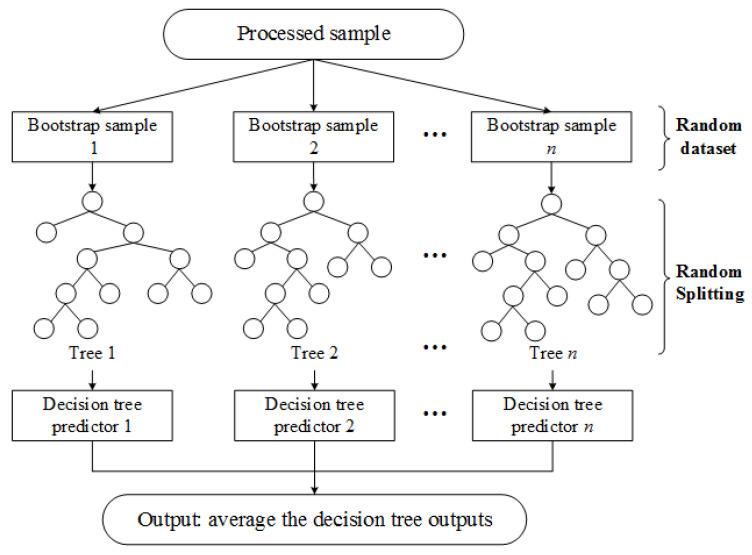
The schematic diagram of random forest regression.

## Data Availability

Data is contained within the article or supplementary material.

## Supplementary Materials

The following are available online at https://www.mdpi.com/1996-1944/14/5/1068/s1, the dataset used to train the ML model.

## Appendix A. Detailed Introduction to the RF Modeling Technique

Random forest, proposed by Breiman and Cutler in 2001 [42], is an ensemble-learning algorithm based on tree predictors. It uses the bootstrap resampling approach to extract samples with replacement from the original training set, conducts decision tree modeling for each bootstrap sample, and then aggregates the prediction outputs of multiple decision trees. When RF is used for regression problems, the final output of the regression is the average of results produced by decision-tree predictors. As displayed in Figure A1, each tree in the “forest” is grown with a randomized subset. Some data may be used more than once in the training, while other may never be used. Moreover, each split at each tree node is created based on a randomly selected subset of input features. The introduction of two randomness increases the diversity of the trees. Thus, greater stability or less overfitting of RF is achieved due to the randomness, which makes RF more robust when there are slight variations in input data [42]. Another advantage is the immunity to noise, since non-correlated trees are generated through different training samples. A weak predictor may be sensitive to noise, but the average of several decorrelated decision trees can largely decrease the noise sensitivity [43]. Compared with the traditional machine learning methods, such as artificial neural networks, RF is easy to train and has fewer parameters. In addition, the feature importance can be identified by out-of-bag samples, which are not used in the RF training process .
